# Asymmetric ommatidia count and behavioural lateralization in the ant *Temnothorax albipennis*

**DOI:** 10.1038/s41598-018-23652-4

**Published:** 2018-04-11

**Authors:** Edmund R. Hunt, Ciara Dornan, Ana B. Sendova-Franks, Nigel R. Franks

**Affiliations:** 1 School of Biological Sciences, University of Bristol, BS8 1TQ Bristol, UK; 2 Department of Engineering Design and Mathematics, University of the West of England, BS16 1QY Bristol, UK

## Abstract

Workers of the house-hunting ant *Temnothorax albipennis* rely on visual edge following and landmark recognition to navigate their rocky environment, and they also exhibit a leftward turning bias when exploring unknown nest sites. We used electron microscopy to count the number of ommatidia composing the compound eyes of workers, males and queens, to make an approximate assessment of their relative sampling resolution; and to establish whether there is an asymmetry in the number of ommatidia composing the workers’ eyes, which might provide an observable, mechanistic explanation for the turning bias. We hypothesise that even small asymmetries in relative visual acuity between left and right eyes could be magnified by developmental experience into a symmetry-breaking turning preference that results in the inferior eye pointing toward the wall. Fifty-six workers were examined: 45% had more ommatidia in the right eye, 36% more in the left, and 20% an equal number. A tentative connection between relative ommatidia count for each eye and turning behaviour was identified, with a stronger assessment of behavioural lateralization before imaging and a larger sample suggested for further work. There was a clear sexual dimorphism in ommatidia counts between queens and males.

## Introduction

Organisms must acquire and use information from the environment to make critical decisions such as where to move to find food or safety, or to find potential mates. Therefore, an examination of an animal’s sensory organs may indicate the sorts of information it needs to acquire, and by association the sorts of behaviours in which it typically engages. At the same time, evidences of left-right asymmetries in the nervous system and behaviour of invertebrates are becoming increasingly common^1–3^, including in red wood ants *(Formica rufa*) engaging in trophallaxis^4^ and ‘rock ants’ *(Temnothorax albipennis*) scouting unfamiliar nest sites^5^. Asymmetries in behaviour are thought commonly to originate in asymmetries in the body, typically the nervous system: in vertebrates there are several examples in birds^6,7^. However, relatively little work has been done to correlate behaviour with readily observable morphological asymmetries in the visual system, a crucial sensory modality for many organisms. Letzkus *et al*. found lateralization of visual learning in the honeybee, and proposed a precise comparison of left-right ommatidia numbers^8^, which we carry out here for an ant species. A detailed study of the honeybee ommatidial array found the average ommatidia count for 7 eyes from 6 bees was 5,432. It compared the left and right eyes of one bee and intriguingly found a difference of 48 ommatidia between right and left^9^, though without reporting which eye had more ommatidia, while Letzkus *et al*. found better learning of a colour stimulus with the right eye^8^. Differences in ommatidia count between left and right eyes of around 1–3% were also found in a study of flies of 5 different species^10^. Other sensory systems, notably olfaction, have been studied in detail in the honeybee in relation to lateralization^3,11–16^. In vertebrates, several studies by L. Rogers correlate asymmetries in the anatomy of the visual system with behavioural asymmetries in birds^17–19^. Another recent study of cockatoos (parrots in the bird family Cacatuidae) found significantly higher ganglion cell densities in the left (52,000–72,000 cells/mm^2^) compared with the right (42,500–50,000 cells/mm^2^) perifoveal region in species that have strong left eye–left foot lateralized behaviours; no such differences are found in species without such lateralized behaviours^20^. In invertebrates other than insects, a study of the common cuttlefish, *Sepia officinalis* (a cephalopod mollusc) found that, the larger the right optic lobe and the right part of the vertical lobe, the stronger the bias to turn leftwards^21^. In ‘cockeyed’ squids, of the family Histioteuthidae, the left and right eyes are dimorphic in size, shape and sometimes lens pigmentation: the large eye is oriented upward to view silhouetted objects, and the smaller eye slightly downward to view bioluminescent point sources^22^. On the other hand, a study of male tarantulas (*Brachypelma albopilosum*) found a rightward lateralization in T-mazes, but could not identify any external morphological differences between left and right eyes, lengths of the first legs or densities of mechanoreceptors and chemoreceptors on the tarsi of males’ first legs, which might indicate perceptual asymmetry^23^. Here, we present the first study to investigate a link between externally observable asymmetries in compound eyes and lateralization in insects. Specifically, we investigate differences in sampling resolution indicated by differential ommatidia count in left and right eyes in worker ants, and their potential correlation with ants’ behavioural choices.

## Compound eye quality and ecological context

Workers of the house-hunting rock ant *Temnothorax albipennis* use edge following for visual navigation, and also respond to fixed landmarks^24,25^. *T. albipennis* have apposition compound eyes composed of ommatidia, and have previously been conservatively assumed to have a 7 degree angle of acuity (spatial resolution)^25^. Recent work on *Temnothorax rugatulus* reports an average inter-ommatidial angle of 16.8 degrees in workers, suggesting a considerably poorer spatial resolution than this assumption^26^. Ommatidia are the units of compound eyes, comprising a lens, receptors and other structures. The capacity of compound eyes to resolve fine detail depends on the number of ommatidia, with the number of ommatidia determining the sampling resolution of ants’ eyes; the final spatial resolution depends also on the optical resolution of each ommatidium^27^. We begin an investigation of observable morphological asymmetry by counting the number of ommatidia in both left and right eyes. This is notwithstanding the fact that the overall acuity of an ant’s visual system also depends on factors other than ommatidia count, including interommatidial angle, facet sizes and rhabdom width (the light sensitive part of the photoreceptor) in the compound eye^28,29^. Such factors were examined recently in the robber fly *Holcocephala fusca*^30^. The field of view of the entire eye may also be different between left and right. For example, the geometry of the eye (ray of curvature) also affects the field of view and hence the overall acuity^29^. Asymmetries in the ants’ antennae, which are used for olfaction^31^, may also play a role in lateralized behaviour. For example, honeybees were found to recall olfactory memories better when presented to the right antenna, while the right antenna surface was also found to have more putative olfactory sensilla^13^.

The size of an organism’s organs are observed to scale with available resources while maintaining function; insect compound eyes are an exemplar of this phenomenon^32^. Therefore, we expect some variation in ommatidia count in different worker ants and different colonies owing to the resources available in their environment during development. A relationship between worker size and ommatidia has been identified in the ant *Camponotus pennsylvanicus*, with larger workers having more ommatidia in their compound eyes^33^. It was also found that reproductives have a much larger number of ommatidia, as they must conduct mating flights, but there was no significant difference between counts in males and females^33^. Behavioural variation in workers was hypothesised to be correlated with a variable number of ommatidia, controlled via development through variable feeding of larvae^33^. Such a correlation has been found in the Sahara desert ant *Cataglyphis bicolor*, where the smallest worker ants inside the nest have 600 ommatidia, whereas the largest, the hunters, have up to 1200^34^. Larger workers were found to orientate more efficiently than smaller workers^35^, consistent with them having better vision from more ommatidia. A positive correlation between body (head) size and ommatidia count has also been found in the Australian desert ant (*Melophorus bagoti*)^36^, and between body (femur) size and compound eye facet diameter in the wood ant (*Formica rufa*)^32^. The scaling rules governing wood ant compound eye structure have been found to differ by colony, which emphasises the sensitivity of organismal development to prevailing environmental conditions, even in a local area^37^. A study of four species of Australian *Myrmecia* ants found caste-specific differences in compound eyes depending on the species’ temporal niche, i.e. different lighting conditions: facet number, facet size, and rhabdom width were all larger in nocturnal castes^38^. The size of ant eyes is also positively correlated with the size of the optic lobes in their brain, commensurate with the greater information processing demands resulting from larger eyes^39^. Vision has both a spatial and temporal component, and thus information processing demands also originate in the movement of eyes over time, which are critical to what information may be collected. For light, manoeuverable insects, movement can closely track visual goals, as in flight^28^ or performing saccade-like body turns during retinoscopic matching^40^. For *Temnothorax*, worker movement is relatively slow and eyesight relatively poor, hence less visual information is likely to be collected than in many social insect species, but worker visual perceptions are still likely to have considerable influence over behaviour^25^. At the same time, ant species with small colony sizes like *T. abipennis* (up to 100–400 workers^41^) typically have a female calling mating system whereby males fly to the queens for matings^31^. Thus, we might expect males to have a higher ommatidia count than queens or workers to facilitate visual goal seeking in flight. In sum, then, we expect variation in *T. albipennis* ommatidia count owing to differences in body size (though no correlation was found with head width in *T. rugatulus*^26^), and variation due to reproductive caste (worker, queen or male) which has not, until now, been investigated in this genus.

### Behavioural lateralization in *T. albipennis*

*T. albipennis* have been found to exhibit a leftward turning bias when exploring unfamiliar nest sites^5^. Hunt *et al*. described two experiments: the first observed ants entering a rectangular nest cavity where 35/89 (39.3%) went left, 19/89 (21.3%) went right and 35/89 (39.3%) went straight on, or 64.8%/35.2% left/right excluding straight on; in the second, ants entered a branching cavity and thus had to choose left or right, and 50/80 (62.5%) went left at the second bifurcation, while 30/80 (37.5%) went right. *T. albipennis* may also prefer to view landmarks with their right eye^42^. Such motor and sensory lateralizations are typically associated with asymmetries in the nervous system^1^. One possible explanation for the leftward turning bias is a sensory lateralization to prefer viewing potential predators with the left eye. This has been observed in fish and lizards, for example^43,44^. Sensory lateralizations have also been observed in many species of mammals, amphibians and birds, with domesticated hen chicks (*Gallus gallus domesticus*) being studied particularly extensively^7^. As the ants enter an unknown and potentially dangerous space, this could bias them to prefer left turns, all else being equal; there have been observations across multiple ant species of disproportionate appendage severance on the left in interspecific fights, and a greater propensity to turn left when alarmed^44^. However, another explanation for a leftward bias may be an asymmetry in the visual system whereby the ants have slightly better vision in one eye than the other, and hence prefer to point their inferior eye toward the wall as they turn, keeping the superior eye facing outward. For instance, a left-turning ant may have slightly better vision in its right eye. Even a tiny asymmetry may have an important influence on behaviour in contexts where two directional choices seem identical, breaking an otherwise symmetrical preference, especially when the accumulation of experience in such situations is considered. It may be that *T. albipennis* workers have one eye with slightly more ommatidia than the other, and that this is associated with marginally better vision on one side of an ant’s visual field, providing a mechanistic explanation for the turning bias. As noted, the acuity of an ant’s visual system depends on factors other than ommatidia count. Here, though, we focus on ommatidia count asymmetries between left and right as a starting point.

In the present paper, we investigate the size of *T. albipennis* worker, queen and male eyes with the goal of: (i) assessing their relative spatial resolution and hence their behavioural roles in the colony; and (ii) identifying a possible link between lateralization of behaviour in worker ants (turning biases when exploring unknown nest sites) and asymmetry in their left and right eye ommatidia counts.

## Materials and Methods

### Ant colonies and sample selection

**Figure 1:**
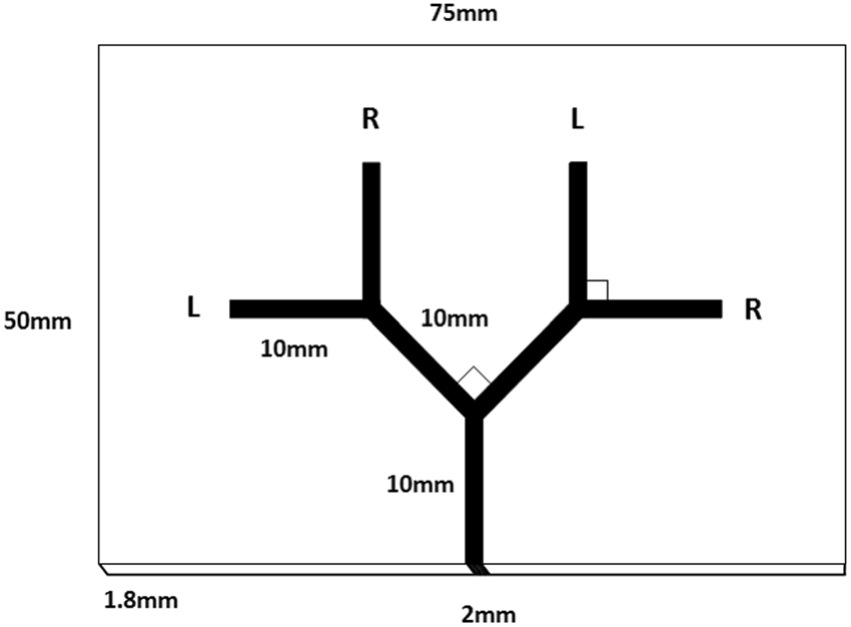
The branching nest site used to select ‘left-biased’ and ‘right-biased’ ants (see also Hunt *et al*. 2014)

We examined 4 colonies of *Temnothorax albipennis* collected from Dorset, UK in August 2014. Following the same branching nest cavity design as in our previous experiment on lateralization of ant exploration behaviour^5^ we selected 7 ants that went down the left branches, and 7 that went down the right (Fig. 1) for each colony. After the colony’s nest had been destroyed, scouting ants looked for a replacement nest site, and after a single ant entered the branching nest cavity we observed its turning choices and placed it into one of two separate Petri dishes using entomological forceps, for those that had chosen left or right in the second branch. The statistical analysis for Hunt *et al*. indicated that thigmotaxis (wall-following) did not influence the choice at the second branch^5^. The ants had no prior experience of the branching nest site. A replacement new nest site was immediately introduced to prevent any influence of olfactory cues from previously exploring ants. The process continued until at least 7 ants were picked for each direction. Care was taken to ensure constant laboratory lighting conditions and the absence of any confounding landmarks; ants were selected in one session, and then euthanized by freezing. Each ant was only tested once to achieve a timely sample selection; as noted in the Discussion, future research would benefit from multiple testing, where possible, to obtain a laterality index for each individual subject. However, the approach taken here allows lateralization to be assayed within the context of a natural collective colony behaviour (searching for a new nest site) which makes the study more relevant to the study species. It is also worth noting that a one-off individual assay leads to noisier behavioural data, and thus makes the establishment of a statistical relationship between turning direction and asymmetric ommatidia count more demanding.

This procedure led to a sample of 56 worker ants, or 112 worker eyes. Colonies 1, 2 and 3 had a queen, with colony 1 additionally having 5 virgin queens. We took a further 10 queens from another 10 colonies not used in our experiment that had been previously euthanized, for a sample of 18 queens. Colonies 1, 2 and 3 had 9, 4 and 1 males respectively, which were all imaged for a sample of 14 males (Table 1).

**Table 1:** The size of sample colonies (a further 10 colonies provided an additional 10 queens).

Colony	Number of workers	Number of brood items (estimate)	Queenright	Virgin queens	Males
1	141	115	Y	5	9
2	202	132	Y	0	4
3	111	48	Y	0	1
4	172	141	N	0	0

### Imaging procedure and data acquisition

Once the sample ants had been euthanized, to facilitate laying their eyes flat for ease of imaging, we sectioned their heads into halves using a scalpel and an optical microscope (60 × magnification). We then fixed them to a sample stub 1 cm in diameter with an adhesive dot. After sputter coating the sample with a thin layer of conductive gold-palladium alloy we imaged the samples with a Quanta 400 Tungsten Scanning Electron Microscope (SEM). Workers were imaged at 600 × magnification and queens and males at 500 × magnification because their eyes were larger. Images were saved as 1024 × 943 pixel, 170dpi TIF files. The number of ommatidia composing the ant compound eyes were determined through manual inspection of the images using the ImageJ counting tool. For worker eyes it was possible to obtain an accurate count of the ommatidia but it was more challenging to do this for the queens and especially the male eyes. This is because they were much larger and more bulbous, and hence a proportion of the ommatidia were out of the field of view. As such, counts were not obtained with sufficient confidence to assess whether there was a left–right difference between eyes for queens or males. For the male ants, the minimum (visible and counted) ommatidia numbers are reported, as well as an estimate of the true numbers. Future work could establish these numbers more precisely by combining multiple counts from different angles. In this first investigation, a rough estimate was sufficient to indicate caste differences in overall ommatidia counts.

### Statistical analysis

Statistical analysis was carried out in the IBM SPSS statistics software package, version 23. To assess whether there was a difference in the total number of ommatidia (left eye plus right eye) between those worker ants that turned left and right in the lateralization assay, we used an independent samples Mann−Whitney *U* test. To assess whether the total worker ommatidia count differed by colony we used an independent samples Kruskal-Wallis test, and a post-hoc test to identify which specific between-colony differences were significant. To test whether the average number of ommatidia per eye was different between queens and males, we again used an independent samples Mann−Whitney *U* test.

The proportion of ommatidia in the left eye was used as a measure of the asymmetry between an individual worker ant’s eyes. This measure was found to be normally distributed (Shapiro-Wilk: *p* = 0.366). A generalized linear mixed model (GLMM) was constructed with direction turned as the response variable and the proportion of total ommatidia on the left eye as the fixed predictor. A binomial response was specified (since the ants could choose either left or right) and a logit link used. Colony and ant were included as a random effect, with ant identity as a factor nested within colony. One ant (ID 48) was identified as an outlier in its proportion of total ommatidia in the left eye, and so the GLMM was estimated both including and excluding this data point.

### Data Availability Statement

The ommatidia count data analysed here is available online at 10.17632/dfwwjmtmfh.1.

## Results

**Figure 2:**
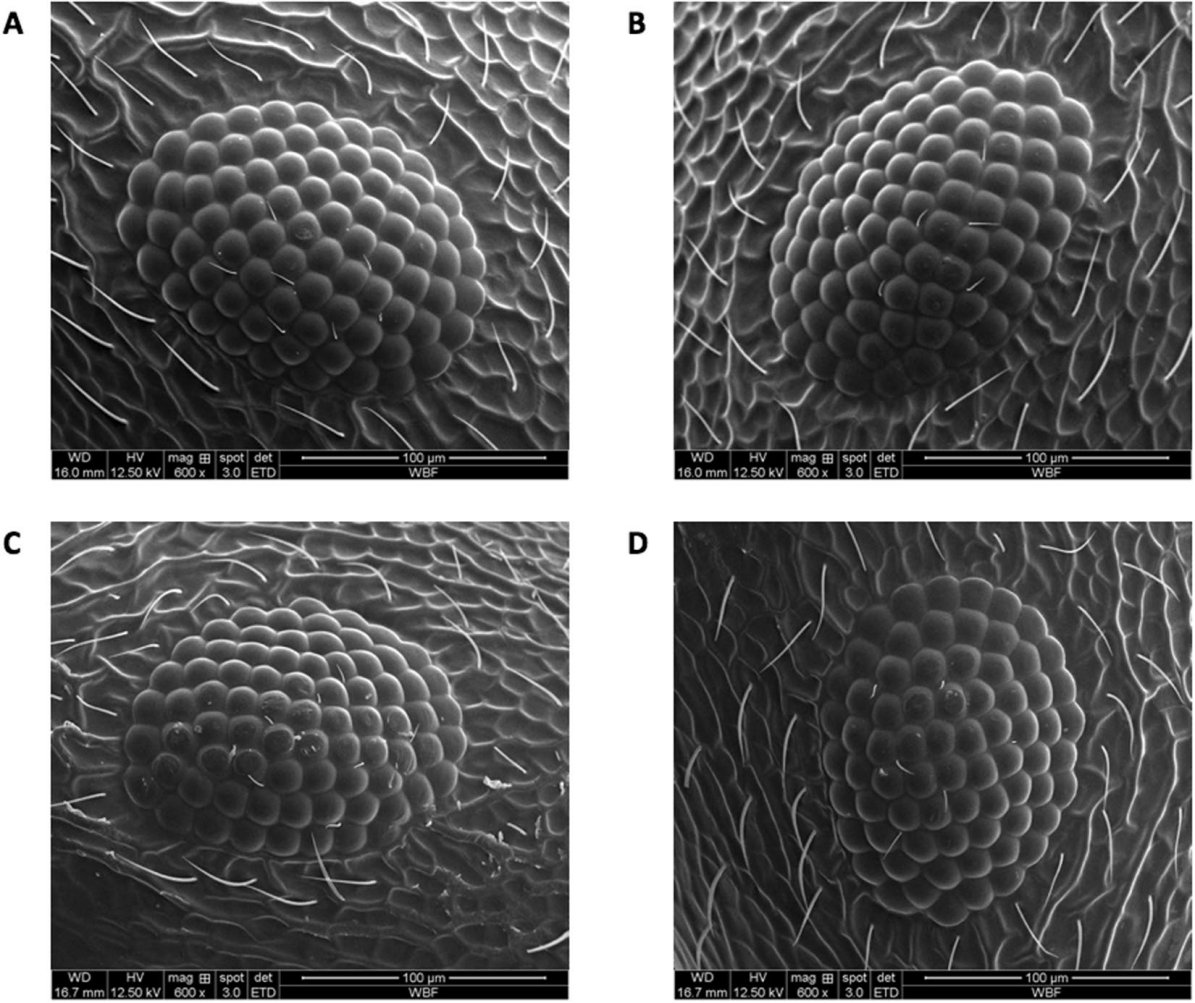
Example SEM images of colony 1 workers for the two branch choices. 600 × magnification. (**A**) Left turn, left eye: Om. = 78. (**B**) Left turn, right eye: Om. = 80. (**C**) Right turn, left eye: Om. = 76. (**D**) Right turn, right eye: Om. = 72.

**Figure 3:**
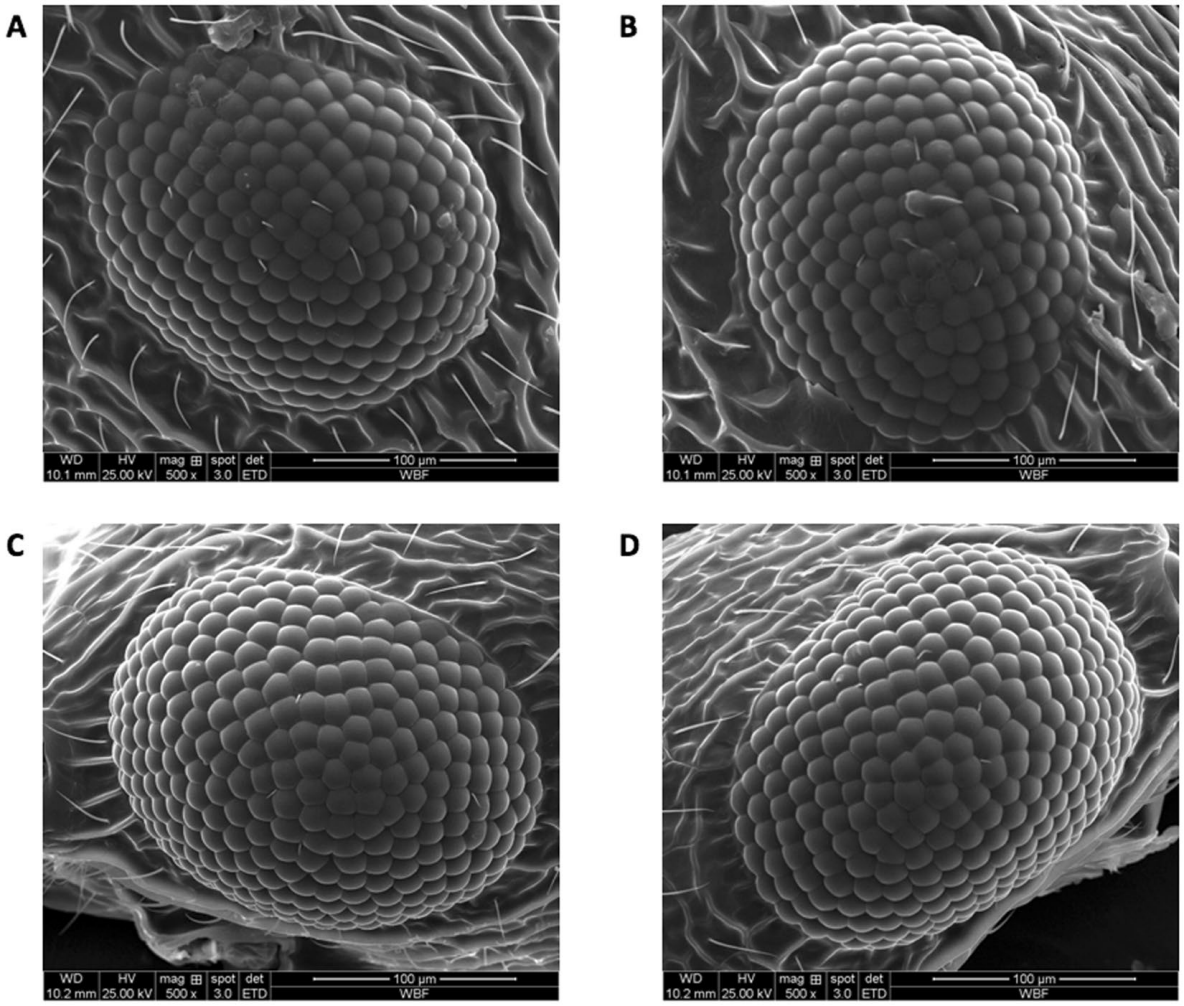
Colony 1 queen and male. 500 × magnification. There is significant uncertainty (estimated to be 10–15%) for the ommatidia counts from the male eyes, since it is difficult to identify them all from one image (time permitting in future research will allow combination of multiple images). We use a minimum count (visible only) for statistical purposes. (**A**) Colony 1 queen, left eye: Om. = 192. (**B**) Colony 1 queen, right eye: Om. = 187. (**C**) Colony 1 male, left eye: Om. (counted) = 274, estimate: 300. (**D**) Colony 1 male, right eye: Om. (counted) = 281, estimate: 310.

Example SEM images of worker eyes (one ant from each of the left/right treatments) can be seen in Fig. 2. Images from a queen and a male are shown in Fig. 3. A slight asymmetry between eyes was typical in the workers: for instance, Fig. 2 shows a difference of *L* − *R* = −2 ommatidia in the left turning ant, and *L* − *R* = +4 in the right turning ant. Expressing the asymmetry as a percentage of the total on the left, to take into account variation in ant size and with 50% being symmetrical eyes, this is 78/158 = 49.3% and 76/148 = 51.4% respectively.

### Ant workers

The mean of the average number of ommatidia in a single eye (i.e. for one ant, the average between left and right) is shown for each of the 4 colonies in Table 2. The overall mean ommatidia number per eye is 77.6 ± 7.0 (standard deviation) for workers.

**Table 2:** Mean of the average (left and right eye) worker ommatidia counts for the 4 colonies.

Colony	Mean	Std. deviation	Minimum	Maximum
1	82.3	6.0	72.5	95
2	76.6	5.3	67.5	90.5
3	71.2	4.6	62.5	82
4	80.4	6.2	70.5	91.5
Total	77.6	7.0	62.5	95

**Figure 4:**
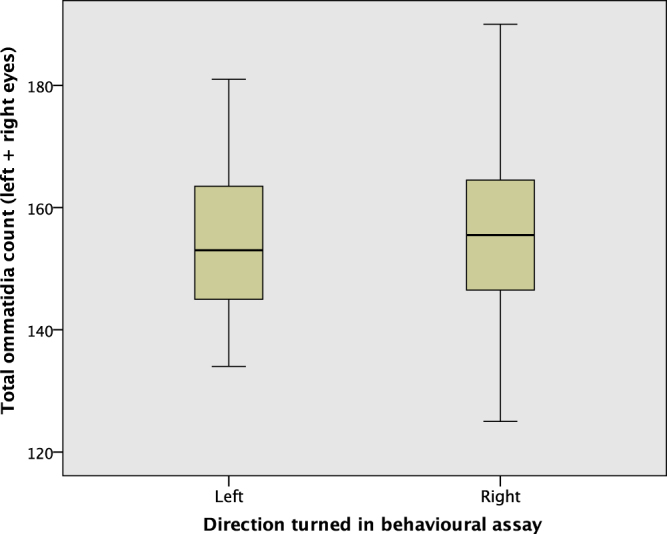
The total number of ommatidia (left plus right), by direction turned. The medians are not different for the two directions.

**Figure 5:**
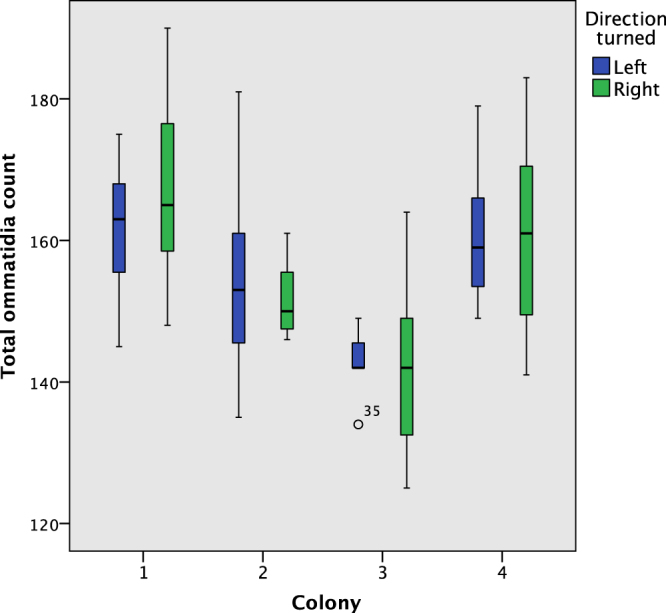
The total ommatidia count by colony and direction turned. Colony 3 has a lower total ommatidia count than 1 and 4.

The overall ommatidia count by direction turned is shown in Fig. 4; an independent samples Mann−Whitney *U* test indicates they have the same median (*p* = 0.902). There is however a statistically significant difference between the distribution of the total ommatidia count across the 4 colonies, as determined by an independent samples Kruskal-Wallis test (*p* < 0.001). Figure 5 shows the total ommatidia count by colony. A post-hoc test showed that colony 3 had a lower average ommatidia count than colony 1 (p < 0.001) and colony 4 (p = 0.002).

**Figure 6:**
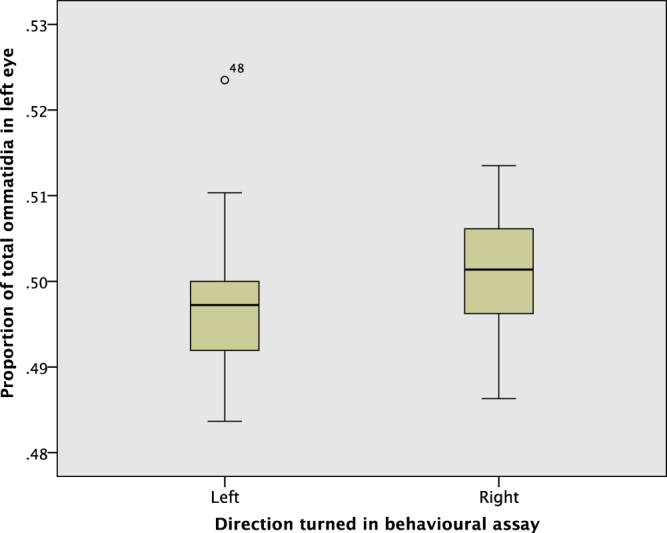
The proportion of total ommatidia on the left eye, by direction turned. Ant 48 (Colony 4, left turn) is an outlier, with 78 ommatidia in its left eye and 71 in its right (52.3% on the left).

**Figure 7:**
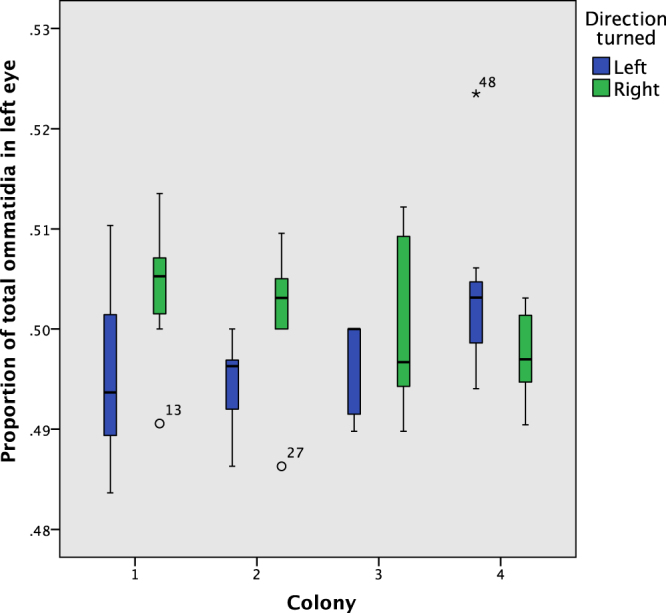
The proportion of ommatidia in the left eye, by colony.

Figure 6 shows the proportion of total ommatidia in the left eye by direction turned. Ants that turned left tended to have more ommatidia in their right eye, whereas ants that turned right tend to have more in their left. Perhaps surprisingly, an identical number of ommatidia in the left and the right eyes (i.e. 50% of the total in the left eye) was the exception rather than the rule: 25 of 56 (44.6%) had more ommatidia in the right eye, 20 (35.7%) more in the left, and 11 (19.6%) had an equal number. Of these 11 ants with an equal number of ommatidia on each side, 7 chose left, which according to a binomial test, is not a significant bias one way or the other (2-tailed p = 0.549, though N = 11 is small for such a test). Figure 7 shows the ommatidia proportion split out by colony, with a clear difference between left and right in colonies 1 and 2 (more ommatidia in the left eye for right-turning ants), and a weaker trend in colony 3, whereas colony 4 has the trend in the opposite direction. Ant 48, a left turning ant from colony 4 and marked as an outlier in Figs 6 and 7, is particularly influential in the result. As an outlier from observations made in the other 55 ants the generalized linear mixed model (GLMM) analysis was run both including and excluding this data point.

When ant 48 was retained in the dataset the proportion of ommatidia on the left eye was not quite significant as a fixed factor explaining directional choice taken (p = 0.123). The random factor colony(ant) (i.e. ant nested within colony) was not significant (p = 0.872). However, when ant 48 was removed from the analysis as an outlier, ommatidia proportion became a significant factor (p = 0.036) in predicting turning direction, with colony(ant) remaining non-significant (p = 0.847). Specifically, the proportion of ommatidia in the left eye had a positive effect direction, where a right turn was coded as 2 and left as 1, indicating a greater likelihood of turning right when ants had more ommatidia in the left eye. This analysis indicates that there may be an effect of the relative size of an ant’s eyes on the direction of its movement in certain contexts.

### Ant sexuals (males and queens)

**Figure 8:**
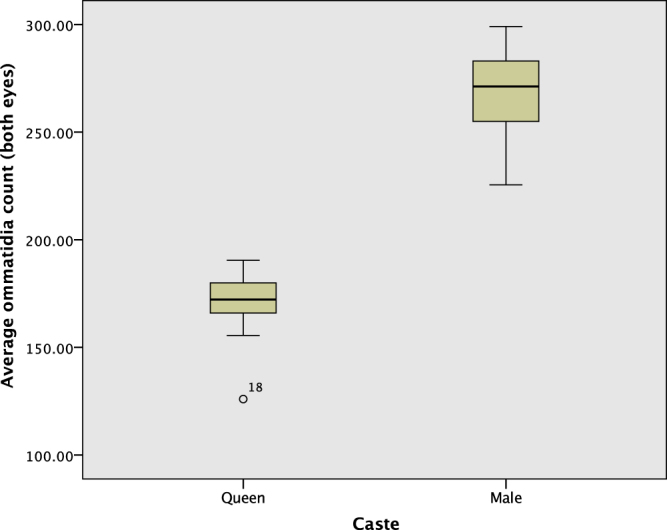
The ommatidia count per eye for the queen and male caste. The male count is a minimum, and is an underestimate of around 10% of the true total.

**Figure 9:**
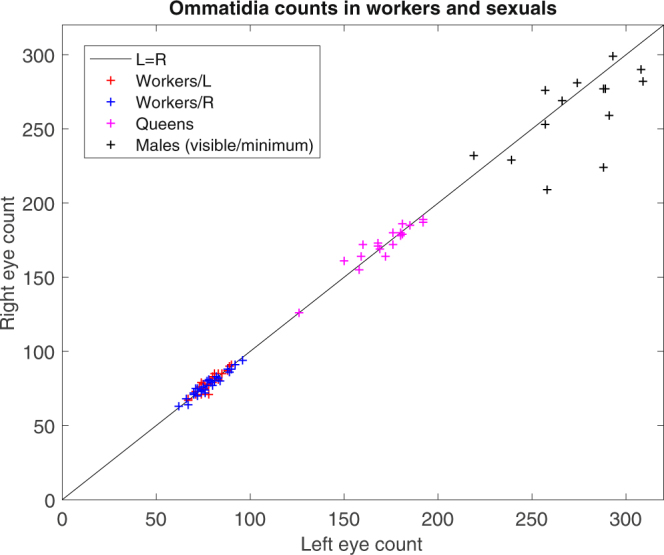
The ommatidia count for the three castes in *Temnothorax albipennis*: workers, queens, males. Workers/L and /R refers to workers that turned left or right. The male ommatidia counts are estimates and hence an asymmetry is not established. We estimate the true mean for males is around 300.

Since there is considerable uncertainty in the ommatidia count for males, because it was difficult to count them all from one image, we consider the minimum (visible) number for statistical comparison with the queens. The mean of the average (both eyes) visible ommatidia for males (267.6 ± 23.2, *n* = 14) is significantly higher than the mean count for queens (171.2 ± 14.8, *n* = 18) (*p* < 0.001, Mann−Whitney *U* test). Since the average for males is a minimum (only visible ommatidia were counted), and the true average is estimated to be closer to 300, males are found to certainly have a higher average number of ommatidia than queens, around 1.75 times as many on average (Fig. 8). The ommatidia counts for each of the three castes – workers, queens and males – is shown in Fig. 9, with deviation from the straight line $R=L$ indicating an asymmetry in ommatidia count. The separate ranges they occupy is apparent, which may be associated with separate behavioural requirements (foraging and flight).

## Discussion

Our results provide tentative indication that behavioural lateralization may be predicted from observable morphological asymmetries in compound eye visual systems, that have not, until now, been considered. Our data further indicate that there is significant variation in ommatidia number in *T. albipennis* castes (Fig. 9), which may relate to differing roles within the colony. In the worker ants, having an identical number of ommatidia in the left and right eyes was the exception rather than the rule: only around 20% of worker ants had an identical number. Yet, the differences in number are only a small percentage (1–2% deviation from 50% on one side) and may be understood as natural developmental variation, with insufficient selection pressure for an identical number in each eye. Of course, it must be stressed that evidence for asymmetry at the level of the peripheral nervous system does not exclude the possibility that asymmetry at a central level could also be observed in future research. For example, a population-level left–right asymmetry in the honey bee’s primary olfactory centre, the antennal lobe, has been observed^45^, following research that found more sensilla, the olfactory structures on the antenna, in each segment of the right antenna^13^. The impact on individual visual perception of the eye asymmetry could be estimated using a model of the ants’ optical system in future research. It may also be possible to identify a more obvious asymmetry by examining species with a higher ommatidia count than the roughly 70 found here; for example bull ants of the genus *Myrmecia* have around 3,200 ommatidia^39^. Nevertheless, we find some tentative evidence for an association between eye morphological asymmetry and turning preference: after excluding a single statistically outlying data point, we found a significant association (*p* = 0.036) between the direction of asymmetry between left and right eyes and the direction turned by the exploring worker ants. While an asymmetry in ommatidia count may have little functional significance, individual experience and social interactions may amplify marginal differences in visual acuity into an observable lateralization of behaviour. The ommatidia count data presented in Fig. 7 shows colonies 1 and 2 exhibit a notable association between turning direction and asymmetry, while it is weaker in colony 3 and weaker, but in the opposite direction, in colony 4. This suggests the possibility that lateralization occurs at the colony-level, rather than the population-level. Colony-level lateralization, as opposed to population-level lateralization, has been identified experimentally for the first time in red wood ants, *Formica rufa*^46^ and predicted in theoretical models which provide for the benefit of within-colony coordination through alignment of individual lateralization^47^. To confirm such an effect would require multiple re-testing of individuals, to establish first the strength of individual lateralization within colonies, before comparing across colonies. It is also the case that such colony-level lateralization may have a social component, rather than being entirely dependent on individual morphology. A large sample within a colony may identify a definitive connection between ommatidia count and turning behaviour, even if the asymmetry between eyes is relatively small, but a large sample across multiple colonies may not if colony is not accounted for as a factor. The indicative trend was for ants that turned right to have more ommatidia in their left eye, and those that turned left to have more ommatidia in their right eye. This trend could be explained as resulting, in the case of a left turning ant for example, from better vision in the ant’s right eye, and hence a preference to obscure vision on the left where necessary by keeping it closer to the wall. As a result, in situations when faced with a decision about which direction to turn in the absence of any environmental cues, a left turn may be preferred (especially when in an unfamiliar and potentially dangerous place). Indeed, of the 56 worker ants examined, 45% had more ommatidia in the right eye, and 36% more in the left, and a left-turning bias has been identified in previous research^5^. The outlier data point, ant 48, confounded this trend by turning left but having 78 ommatidia in its left eye and 71 in its right (52.3% on the left). However, there is likely to be intra-individual variation, i.e. the same ant may turn both left and right with some degree of randomness in identical contexts. In practice, this means that a one-off behavioural assay results in statistical tests of correlation between asymmetric ommatidia counts and lateralization erring on the conservative side. Further research may establish a more definitive connection between ommatidia count and turning behaviour by taking multiple measurements from the same individual to measure a laterality index ${LI}=\frac{\text{(right turns)}-\text{(left turns)}}{\text{Total turns recorded}}$ which varies between −1 (always turns left), 0 (turns left and right in equal proportion) and + 1 (always turns right). The one-off behaviour test employed here is unlikely to always assign lateralized ants to the appropriate group of ‘left turners’ and ‘right turners’. For instance, an ant with a 60% left-lateralization will tend to make right choices 40% of the time, and a few such misassignments will weaken the correlation with asymmetric ommatidia counts, if such an effect exists. However, once a laterality index occupying a continuous scale has been measured with some degree of confidence (after at least 10 observations,) the ant could be imaged and the index included as a fixed effect in a GLMM analysis. Observations could be done using individual paint markings and repeated over a period of days so that the effect is not confounded somehow by an ant’s memory of the laterality test. However, there are costs to performing repetitions of the biologically-relevant behavioural assay employed here, because it requires repeatedly forcing ants to leave their home nest to search for a replacement nest site, and waiting for the same, previously marked scouting ants to enter another new nest site. A more easily repeatable assay could involve observations of turning preference inside larger branching mazes, for example, but it would be less relevant to the natural colony emigration process, where scouting ants are motivated to explore. Lateralization can also be measured through preferential forelimb use in crossing gaps in walkways, as for example in the work of Bell studying the red wood ant *Formica rufa*^46^, and in studies of the desert locust, *Schistocerca gregaria*^48,49^. Interestingly, Bell did not find any correlation between individual *Formica rufa* worker’s turning preference and forelimb preference^46^ (N = 19). Such intra-individual variation in lateralization has also been observed in fruit flies (*Drosophila*), which exhibit both individual-level lateralization in turning and wing-folding behaviour, but without correlation between the two^50^. This suggests different neuronal components are responsible for different lateralized behaviours^46^. Therefore, future investigation of potential associations between morphological asymmetries and lateralization could consider a variety of relevant behavioural indicators.

Our investigation of caste differences (worker or reproductive) in ommatidia count found substantial variation. There are a significantly higher number of ommatidia in the male ants’ eyes (measured minimum mean 267.6 ± 26.6, estimated mean $\approx 300$) compared to the queens’ eyes (mean 171.2 ± 15.1) (*p* < 0.001). The increased energy cost associated with such a larger number of ommatidia compared to the workers suggests that it confers a significantly higher probability of mating success. The difference compared to queens and workers is also especially striking considering that males have smaller bodies, and suggests that the males may have to find the queens partially using their sight. This is consistent with a female-calling mating system in *Temnothorax albipennis*, or at least a reduced female flight system, which would also conform with the general trend for female-calling in species with small colony sizes^31^. Males would benefit from an enhancement to their vision to identify calling females located on the ground, from the air or once they had landed, presumably in combination with sensing of a pheromone gradient^31^. Male ants, with their much higher number of ommatidia than workers, are likely to have a larger asymmetry between eyes in absolute terms, and thus an association between ommatidia count and lateralization may be more easily determined in this caste.

Here we made a simple assessment of ant visual acuity in left and right eyes using ommatidia counting, and found an asymmetry that was significantly correlated with worker turning direction in branching mazes, as they scouted for a new nest site for their colony. We hope this points the way to further studies of insect compound eye asymmetry and behavioural lateralization: such research has been mooted previously^8^ and ommatidia asymmetries have already been indicated in honeybees^9^ and various flies^10^. As previously noted, while sampling resolution depends on ommatidia count, the overall acuity of the ants’ visual system depends on additional, externally observable factors, including facet sizes and total field of view^28^; these may be further mechanistic factors contributing toward lateralization of behaviour, as well as asymmetries in the ants’ antennae^13^. Future work could include a study of such factors, alongside studies of brain asymmetry^45^, and thus better identify the proximate causes of lateralized behaviour.
